# Scratching the Surface—An Overview of the Roles of Cell Surface GRP78 in Cancer

**DOI:** 10.3390/biomedicines10051098

**Published:** 2022-05-10

**Authors:** Jack Chen, Edward G. Lynn, Tamana R. Yousof, Hitesh Sharma, Melissa E. MacDonald, Jae Hyun Byun, Bobby Shayegan, Richard C. Austin

**Affiliations:** 1 Department of Medicine, Division of Nephrology, St. Joseph′s Healthcare Hamilton, Hamilton Center for Kidney Research, McMaster University, Hamilton, ON L8N 4A6, Canada; chenj63@mcmaster.ca (J.C.); elynn@stjosham.on.ca (E.G.L.); yousoftr@mcmaster.ca (T.R.Y.); sharmh3@mcmaster.ca (H.S.); melmacdo@stjosham.on.ca (M.E.M.); byunjh@mcmaster.ca (J.H.B.); 2 Department of Surgery, Division of Urology, The Research Institute of St. Joe′s Hamilton, McMaster University, ON L8N 4A6, Canada; shayeb@mcmaster.ca

**Keywords:** GRP78, cancer, autoantibody

## Abstract

The 78 kDa glucose-regulated protein (GRP78) is considered an endoplasmic reticulum (ER)-resident molecular chaperone that plays a crucial role in protein folding homeostasis by regulating the unfolded protein response (UPR) and inducing numerous proapoptotic and autophagic pathways within the eukaryotic cell. However, in cancer cells, GRP78 has also been shown to migrate from the ER lumen to the cell surface, playing a role in several cellular pathways that promote tumor growth and cancer cell progression. There is another insidious consequence elicited by cell surface GRP78 (csGRP78) on cancer cells: the accumulation of csGRP78 represents a novel neoantigen leading to the production of anti-GRP78 autoantibodies that can bind csGRP78 and further amplify these cellular pathways to enhance cell growth and mitigate apoptotic cell death. This review examines the current body of literature that delineates the mechanisms by which ER-resident GRP78 localizes to the cell surface and its consequences, as well as potential therapeutics that target csGRP78 and block its interaction with anti-GRP78 autoantibodies, thereby inhibiting further amplification of cancer cell progression.

## 1. Introduction

**Figure 1:**
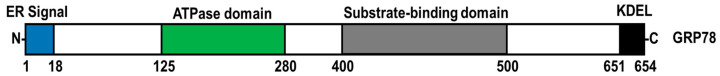
Functional domain structure of GRP78. GRP78 is composed of an ER signal sequence, ATPase domain, substrate-binding domain, and a C-terminal KDEL tetrapeptide sequence.

The endoplasmic reticulum (ER) is a complex organelle responsible for protein synthesis and folding, the storage of intracellular Ca^2+^ and lipid metabolism [1,2]. ER-resident chaperones facilitate the capacity of the ER for protein folding and prevent the aggregation of misfolded polypeptides [3]. Within the ER, the 78 kDa glucose-regulated protein (GRP78, also known as BiP; *HSPA5* gene) assists in folding and the quality control of nascent polypeptides by binding to exposed hydrophobic motifs on misfolded polypeptides in an ATP-dependent manner (Figure 1) [4]. Furthermore, ER-resident GRP78 is a vital modulator of oxidative stress, lipotoxicity, inflammation, ER Ca^2+^ depletion, glucose deprivation, hypoxia and viral infection, which all can disrupt ER homeostasis and lead to an accumulation of misfolded and unfolded proteins, a condition known as ER stress [5,6,7,8,9,10,11]. To mitigate ER stress, GRP78 dissociates from protein kinase RNA-like ER kinase (PERK), inositol-requiring protein 1α (IRE1α) and activating transcription factor 6 (ATF6), provoking a dynamic signaling cascade known as the unfolded protein response (UPR) [10,11]. Activation of the UPR leads to the inhibition of de novo protein synthesis, the degradation of misfolded ER proteins and the upregulation of protein chaperone expression [12]. In cases where ER stress is insufficiently mitigated, chronic UPR activation can lead to the upregulation of proapoptotic signaling [13].

Highly proliferative tumor microenvironments are hypoxic and glucose-deprived, negatively impacting protein folding and resulting in ER stress and elevated GRP78 expression [14,15]. Upregulated GRP78 expression is associated with tumor proliferation, metastasis, neovascularization, and poorer prognosis in cancer patients [16,17]. Although GRP78 has classically been viewed as an ER-resident molecular chaperone, it has been reported in several other cellular organelles, including the nucleus and the mitochondria [18,19]. Additionally, GRP78 has been observed on the cell surface of several human cancers, including prostate cancer, breast cancer, ovarian cancer, brain cancer, melanoma cancer, leukemia and lymphoma, where it functions as a signaling receptor [20,21,22,23,24]. Furthermore, the expression of cell surface GRP78 (csGRP78) induces a humoral response that leads to the generation of anti-GRP78 autoantibodies in patients with cancer, which is associated with disease progression, elevated risk of metastasis, and reduced overall survival [25]. This review discusses the role of csGRP78 and how its interaction with anti-GRP78 autoantibodies contributes to cancer progression. The feasibility of targeting the engagement of this csGRP78/anti-GRP78 autoantibody complex as a therapeutic strategy for the treatment and management of cancer is also examined.

## 2. Mechanisms of GRP78 Translocation to the Cell Surface 

Although early work using prediction software and fluorescence activated cell sorting (FACS) analysis suggested csGRP78 was a transmembrane protein [26], more recently, Tsai and colleagues demonstrated that csGRP78 is a non-transmembrane peripheral membrane protein in several cancer cell lines, as determined by sodium bicarbonate extraction [27]. Furthermore, it was revealed that csGRP78 associates with several membrane proteins, including glycosylphosphatidylinositol (GPI)-anchored membrane proteins [27].

**Figure 2:**
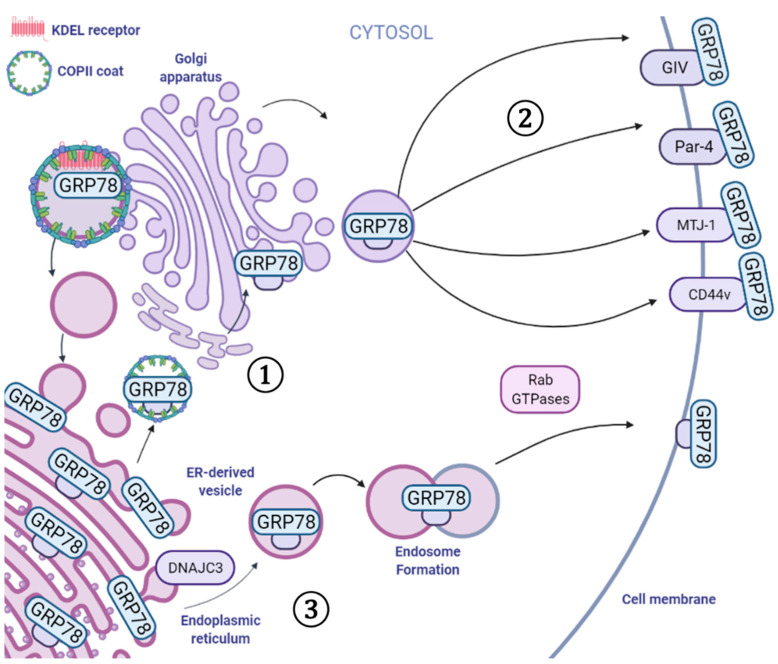
Mediators that facilitate the cell surface localization of GRP78. Under normal physiological conditions, GRP78 is retained in the ER through interactions with the KDEL sequence and the KDEL receptor. ① Under conditions of ER stress, GRP78 can escape ER retention and pass through the Golgi apparatus. ② Moreover, several proteins, including GIV, Par-4, MTJ-1, and CD44v, interact with GRP78 in the ER, which facilitates GRP78 translocation to the cell surface. ③ Alternatively, GRP78 can escape the ER with the assistance of DNAJC3 within an ER-derived vesicle and undergo endosome formation and fusion mediated by several Rab GTPases. Image generated with BioRender.

Under normal physiological conditions, csGRP78 expression has been observed at low levels, which can be exacerbated under conditions of ER stress [26]. Although the exact mechanism by which GRP78 is translocated to the cell surface is yet to be fully elucidated, several mechanisms have been proposed (Figure 2). One potential mechanism of csGRP78 localization involves the KDEL retrieval system [26]. ER-resident chaperones, including calreticulin, protein disulfide isomerase, GRP94, and GRP78, contain a conserved C-terminal KDEL motif that is required for their retention within the ER lumen [28]. KDEL-containing polypeptides are recognized by KDEL receptors (KDELR), located in the cis-Golgi, which facilitate retrograde ER–Golgi trafficking mediated by COP I vesicles [28,29]. Under conditions of ER stress, the elevated expression of KDEL-containing proteins is believed to overwhelm the capacity of the KDELR and allow GRP78 to escape the ER and reach the cell surface [26]. Consistent with this hypothesis, the ectopic overexpression of full-length GRP78 has been shown to elevate csGRP78 expression in the absence of ER stress [26]. Recent evidence has shown that ER stress also activates the proto-oncogene tyrosine–protein kinase (SRC), which results in KDELR dispersion and the disruption of retrograde Golgi transport of GRP78 to the ER [30]. 

An alternative hypothesis is that ER stress may activate cell-type-specific mechanisms that facilitate GRP78 translocation to the cell surface, such as interactions with the membrane and secreted proteins. Previous reports have indicated that mutations in the substrate-binding domain of GRP78 markedly reduced csGRP78 localization in contrast to mutations in the ATPase domain [27]. Similarly, Limso et al. reported that GRP78 binds to the C-terminus of G_α_-interacting vesicle-associated protein (GIV) under conditions of ER stress [31]. Although the exact mechanism by which GIV contributes to csGRP78 localization remains unclear, the knockdown of GIV was shown to attenuate ER stress-induced csGRP78 expression in HeLa cells [31]. In tamoxifen-resistant MCF7 cells, an isoform of CD44 (CD44v) has recently been reported to bind the C-terminal proline-rich repeat of GRP78 and facilitate the transport of GRP78 to the cell surface (Figure 2) [32,33]. Burikhanov et al. demonstrated that under conditions of ER stress, Par-4 and GRP78 form a complex in the ER, where Par-4 assists in the transport of GRP78 to the cell surface in response to tumor-necrosis-factor-related apoptosis-inducing ligand (TRAIL) treatment [34]. Co-chaperones such as murine Dna-J-like transmembrane protein (MTJ-1) also contribute to the localization of GRP78 to the cell surface [35]. Within the ER, MTJ-1 forms a complex with GRP78, regulating the chaperone activity of GRP78 [36]. Furthermore, the knockdown of MTJ-1 attenuates the expression of csGRP78 in murine macrophages, indicating that MTJ-1 has a role in aiding GRP78 translocation to the cell surface [35]. Consistent with these findings, knockdown of the human homologue HTJ-1 has been shown to attenuate the expression of csGRP78 in human pulmonary artery endothelial cells [37]. 

Interestingly, Van Krieken and colleagues recently reported that GRP78 could bypass the Golgi body and be unconventionally shuttled to the cell surface, a process facilitated by Rab GTPases [38]. This recent discovery implicates ER-derived vesicles, generated by endosomal fusion through vesicle SNARE BET1 and target SNARE Syntaxin 13, in the trafficking of GRP78 and other ER luminal chaperones to the cell surface (Figure 2) [38]. Recently, Kim et al. reported that the acetylation status of GRP78 is also important for the ability of GRP78 to localize to the cell surface of cholangiocarcinoma (CCA) cells [39]. ACY-1215, a selective HDAC6 inhibitor, increased the levels of acetylated GRP78 and suppressed csGRP78 localization in TFK-1 CCA cells [39]. Although the mechanism by which GRP78 acetylation inhibits its translocation to the cell surface is yet to be fully described, it is reasonable to hypothesize that GRP78 acetylation could interfere with its interaction with protein binding partners responsible for GRP78 cell surface translocation. These studies illustrate that the mechanism of csGRP78 localization may be cell-type- or tissue-specific, and may correlate with the physiological function of the cells and the type of proteins that interact with GRP78 in the ER and cell surface. 

## 3. Functions of csGRP78 in Cancer 

### 3.1. Cancer Progression

**Figure 3:**
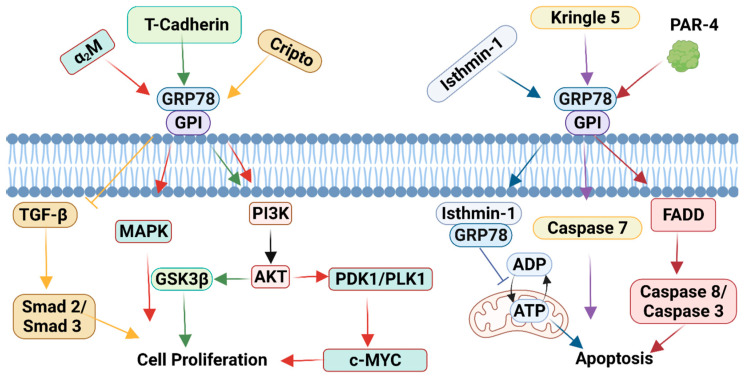
csGRP78 functions as a signaling receptor that mediates cell proliferation or apoptosis. At the cell surface, GRP78 associates with GPI-anchored proteins. Activated α_2_M binds to csGRP78 and activates the PI3K/AKT pathway, which stimulates the PDK1/PLK1 pathway to upregulate c-MYC expression and cell proliferation (red arrows). Similarly, T-cadherin interacts with GRP78 to activate the PI3K/AKT pathway and stimulates GSK3β to increase cell proliferation (green arrows). Furthermore, Cripto binds to csGRP78 and inhibits TGF-β-mediated Smad2/3 activation and results in cell proliferation (yellow arrows). Cell surface GRP78 can also induce apoptosis through isthmin-1 interacting with csGRP78 to allow for GRP78 internalization. This leads to the inhibition of mitochondrial ATP transport and apoptosis (blue arrows). Furthermore, Kringle 5 interacts with csGRP78, which enhances caspase 7 activity (purple arrows). Moreover, Par-4 binds to GRP78 and activates the FADD-induced caspase 8/caspase 3 pathway to lead to apoptosis (maroon arrows). Image generated with BioRender.

The expression of csGRP78 has been reported in several cancers including, but not limited to, leukemia, pancreatic cancer, colorectal cancer, breast cancer, and prostate cancer [40,41,42,43,44,45]. Mechanistically, csGRP78 serves as a multifunctional receptor known to regulate both cell proliferation and apoptotic pathways that contribute to tumor progression (Figure 3) [46]. Cell surface GRP78 was first described as a co-receptor for activated α_2_-macroglobulin (α_2_M), which binds to the N-terminal domain of GRP78 (Leu_98_-Leu_115_) [47]. Previous reports have demonstrated that the binding of activated α_2_M to csGRP78 stimulates the PI3K/AKT and MAPK cell proliferation pathways, promoting tumor cell growth [21,48,49]. Activation of csGRP78 by activated α_2_M has also been shown to promote c-MYC expression by inducing the PDK1/PLK1 signaling axis [50]. The interaction between Cripto and csGRP78 has also been demonstrated to improve cell survival by suppressing transforming growth factor β (TGFβ)-induced Smad2/3 signaling [51,52]. Similarly, csGRP78 has been reported to be complexed with T-cadherin, which regulates AKT/GSK3β signaling and enhances endothelial cell survival [53]. In contrast, binding of plasminogen Kringle 5 to csGRP78 induces endothelial cell and tumor cell apoptosis mediated through the activation of caspase 7 [54,55]. Additionally, csGRP78 is a known receptor for Par-4 and mediator of Fas-associated death domain (FADD)-induced caspase 8 and caspase 3 activation, major components of the extrinsic pathway of apoptosis [34]. Interestingly, the binding of isthmin-1 to csGRP78 was shown to induce csGRP78 internalization, the disruption of mitochondrial ATP transport and apoptosis [56]. Consistent with these observations, isthmin-1 was recently shown to be an important regulator of lung homeostasis by inducing apoptosis in alveolar macrophages expressing high levels of csGRP78 [57]. Furthermore, it was shown that isthmin-1 suppressed 4T1 breast cancer tumor growth and endothelial cell angiogenesis [56].

Cell surface GRP78 has been reported to contribute to tumor cell motility and cell-matrix adhesion through interactions with integrin β1 and FAK1, which are critical mediators of tumor cell metastatic potential [42]. Blocking csGRP78 with an anti-GRP78 antibody was shown to protect against the cell invasion of LoVo, a human colon adenocarcinoma cell line, indicating that csGRP78 may bind to plasminogen to promote tumor cell migration and invasion [42]. A recent study demonstrated that Dermcidin (DCD) acts as a novel binding partner of csGRP78 in breast cancer cells, and that DCD enhanced breast cancer cell migration [58]. Furthermore, Lager et al. demonstrated that overexpressing DCD in MDA-MB-231 breast cancer cells enhanced Wnt signaling, an important regulator of cell migration [58]. 

### 3.2. Chemoresistance and Radioresistance

Cell surface GRP78 expression is also linked to resistance against chemotherapy and radiotherapy. Hormone-therapy-resistant breast and prostate cancer cells have been shown to express elevated levels of csGRP78 and promote PI3K/AKT activation [49]. Similarly, it was reported that csGRP78 protected against radiation therapy by activating YAP/TAZ-mediated gene expression in pancreatic ductal adenocarcinoma cells [59]. One recent study demonstrated that the irradiation of BHY and FaDu head and neck squamous cell carcinoma (HNSCC) cells increased levels of csGRP78 [60]. Overexpression of GRP78 was also shown to provide radioresistance and to enhance migration, compared with vector-control-transfected HNSCC cells [60]. Interestingly, the irradiation of HNSCC cells released GRP78-containing extracellular vesicles and was shown to increase GRP78 levels in non-irradiated recipient cells [60]. Consistent with these findings, it was recently reported that a C-terminal-specific anti-GRP78 antibody-mediated blockage of csGRP78 found on mesenchymal glioma stem cells enhanced sensitivity to radiation therapy [61]. Furthermore, the utility of csGRP78 expression as a diagnostic tool was investigated. Angelez-Floriano et al. recently reported a greater frequency of csGRP78–CXCR4^+^ blood-derived cells in high-risk leukemia patients [45].

### 3.3. Cell Surface GRP78 as an Autoantigen

In 2003, Mintz et al. were the first to identify csGRP78 as an autoantigen that leads to the production of anti-GRP78 autoantibodies in patients with cancer [25]. Patients with early-stage colorectal cancer and polyp patients exhibited elevated levels of anti-GRP78 autoantibodies in sera, compared with healthy control patients [62]. Likewise, autoantibodies against GRP78 were found in the sera of patients with gastric and esophageal cancer [63]. In patients with hepatocellular carcinoma, anti-GRP78 autoantibody titres are associated with clinical stage and tumor metastasis [64]. Remarkably, anti-GRP78 autoantibodies that recognize a CNVSDKSC conformational peptide were identified from a pool of circulating antibodies found in the sera of patients with prostate cancer [25]. The CNVSDKSC conformational peptide was reported to mimic the tertiary structure of Leu_98_-Leu_115_ on GRP78 [25]. Levels of these anti-GRP78 autoantibodies correlate with prostate cancer severity, and levels were dramatically reduced in post-operative prostate cancer patients [44]. Furthermore, the engagement of anti-GRP78 autoantibodies with csGRP78 induces inositol triphosphate (IP_3_)-mediated ER Ca^2+^ release, which is known to promote tissue factor (TF) procoagulant activity, a known contributor of tumor growth and metastasis [44,65,66]. In a separate study, anti-GRP78 autoantibodies also promoted melanoma tumor growth [67]. Mice immunized with full-length recombinant GRP78 (rGRP78) exhibited accelerated B16F1 melanoma tumor growth compared with adjuvant-only control mice [67]. Furthermore, sera from mice immunized with rGRP78 exhibited increased AKT activation in B16F1 cells [67]. In contrast, the effect of sera to stimulate AKT was lost following the depletion of anti-GRP78 autoantibodies [67]. Collectively, this evidence demonstrates that the anti-GRP78 autoantibodies that target csGRP78 found on cancer cells can promote malignant tumor progression.

## 4. csGRP78 as a Therapeutic Target

Targeting tumor-specific surface antigens is a promising therapeutic approach that has been used in anti-cancer treatment due to its potential to mitigate non-specific side-effects associated with contemporary cancer therapies [68]. csGRP78 is expressed predominantly in malignant tumor cells and not normal cells; therefore, targeting csGRP78 can be exploited as a novel therapeutic strategy for cancer treatment. Targeting csGRP78 also has clinical applications, including cancer imaging, which can improve cancer diagnoses. Several promising small peptides, chimeric antigen receptor (CAR) T cells, monoclonal antibodies (MAb), and single-chain variable fragments (scFvs) against csGRP78 have been shown to target tumor cells and attenuate tumor cell progression [69].

### 4.1. GRP78-Binding Small Peptides

The use of small peptides is an innovative approach for the treatment of cancer because short amino acid chains can easily be synthesized with high specificity for cancer-specific targets such as csGRP78 [70]. Arap and colleagues identified two GRP78-binding peptides (WIFPWIQL and WDLAWMFRLPVG) against csGRP78 found on DU145 tumor xenografts [69]. Chimeric fusion peptides of either WIFPWIQL or WDLAWMFRLPVG to the proapoptotic motif _D_(KLAKLAK)_2_ showed a dose-dependent reduction in cell viability in DU145 cells [69]. In support of this concept, both WIFPWIQL-GG-_D_(KLAKLAK)_2_ and WDLAWMFRLPVG-GG-_D_(KLAKLAK)_2_ inhibited DU145 tumor growth in both nude mice and Balb/c mice bearing EF43-*fgf4* breast carcinoma tumors [69]. WIFPWIQL-GG-_D_(KLAKLAK)_2_, later termed bone-metastasis-targeting peptidomimetic-78 (BMTP-78), was further investigated due to its effects in mammary tumor metastasis [71]. BTMP-78 was demonstrated to dose-dependently inhibit the viability of metastatic 4T1.2 breast cancer cells, and such an effect was blocked by treatment with an anti-GRP78 antibody [71]. BMTP-78 also reduced tumor weight in mice bearing 4T1.2 mammary tumors, but did not affect tumor volume in the less metastatic tumor xenografts 67NR or 66cl4 [71]. Notably, BMTP-78-treated mice bearing 4T1.2 tumors exhibited an extended period of disease-free survival compared with mice treated with either saline or control peptide [71]. BMTP-78 was also investigated for its therapeutic use in treating acute myeloid leukemia (AML) [72]. FACS analysis revealed the expression of csGRP78 in mononuclear cells in patients with AML [72]. Moreover, BMTP-78 was demonstrated to dose-dependently reduce cell viability among several human-derived leukemia and lymphoma cell lines and in AML-patient-derived peripheral blood cells [72]. Although this study showed acceptable toxicity levels in small rodents treated with BMTP-78, the use of BMTP-78 in the treatment of AML was halted due to the development of lesions at the injection site, kidney lesions, and cardiac lesions in non-human primates [72]. Furthermore, cardiac arrhythmias were also observed in a separate cohort of female rhesus monkeys intravenously infused with BTMP-78 [72]. 

GRP78-binding peptides may also be utilized as a diagnostic tool in cancer. One previous study examined the use of the GRP78-binding peptide, WIFPWIQL, with radiolabelled polymeric micelles in the nuclear imaging of MKN45 tumor xenografts [73]. Nude mice injected with WIFPWIQL-^111^In-labelled polymeric micelles exhibited elevated radioactive intensity in the tumor compared with mice injected with ^111^In-labelled polymeric micelles alone, demonstrating that targeting csGRP78 can improve cancer imaging. Consistent with these findings, GIRLRG, a synthetic peptide generated based on computational modelling of the ATPase domain of GRP78, was confirmed to bind to GRP78 using surface plasmon resonance [74]. Additionally, the injection of radiolabelled ^111^In-PEG-GIRLRG was shown to enhance the resolution of single-photon emission computerized tomography (SPECT) scans of nude mice bearing either A549 lung, BXPC3 pancreatic, or D54 brain tumors [74].

### 4.2. CAR T Cell Therapy

CAR T cell therapy is a revolutionary cancer treatment approach where patient-derived T lymphocytes are reprogrammed to express synthetically designed receptors that recognize the surface antigens of tumor cells [75]. Hebbar et al. recently generated CAR T cells that specifically target csGRP78 (GRP78.1x, GRP78.2x, and GRP78.3x CAR T cells) found on AML cells [22]. Coincubation of GRP78.1x CAR T cells with MOLM13 human leukemia cells expressing csGRP78 were shown to enhance the levels of anti-tumor cytokines interferon (IFN)-γ, interleukin (IL)-2, tumor necrosis factor α (TNFα), granulocyte-macrophage colony-stimulating factor (GM-CSF), and to a lesser extent, IL-4, IL-5, IL-6, IL-10 and IL-13 [22]. Importantly, all three GRP78–CAR T cell variants were shown to potently suppress MOLM13 xenograft tumor progression in NSG immunodeficient mice [22]. However, the authors indicated that the anti-tumorigenic effect of GRP78 CAR T cells became limited over time due to the depletion of circulating GRP78 CAR T cells [22]. Remarkably, dasatinib-treated GRP78.1x CAR T cells were shown to induce complete remission and extend the overall survival of NSG mice bearing THP-1 tumor cells, compared with mice treated with GRP78.1x CAR T cells alone [22]. The authors indicated that dasatinib prevented early T cell activation by suppressing csGRP78 expression in the CAR T cells, which enhanced GRP78–CAR T cell viability [22].

### 4.3. Anti-GRP78 Antibodies

The use of MAb is an emerging treatment strategy for cancer by targeting tumor cell-specific cell surface proteins [76]. Furthermore, several MAb that recognize csGRP78 have been identified [69]. It is well-established that targeting the C-terminal domain of csGRP78 promotes p53 activation and cell death in cancer cells [77]. Two anti-GRP78 antibodies, C38 and C107, were recently shown to bind to a region near the C-terminus of GRP78 [77]. Furthermore, it was shown that the C38 antibody blocked α_2_M and N-terminal-specific anti-GRP78 antibody-induced activation of AKT [77]. However, the C38 antibody alone did not induce chromatin fragmentation or inhibit B16F1 tumor growth, suggesting that the C38 antibody primarily acts as a steric inhibitor of N-terminal agonists to csGRP78 [77]. In contrast, engagement of the C107 antibody to csGRP78-induced chromatin fragmentation and inhibited B16F1 tumor growth [77]. MAb159 is another C-terminal anti-GRP78 MAb that has been shown to induce csGRP78 internalization and suppress HT29, H249, and A549 tumor progression [78]. Interestingly, A549 tumors treated with MAb159 exhibited enhanced apoptotic TUNEL staining and reduced Ki67 cell proliferation staining [78]. In addition, MAb159 attenuated PI3K activation with a modest effect on ERK1/2 and Src signaling [78]. Moreover, MAb159 suppressed 4T1 tumor metastasis in the liver and lung, compared with IgG-treated mice [78]. 

PAT-SM6 (formerly known as SAM-6) is an anti-GRP78 IgM antibody that was first isolated from a gastric cancer patient [79,80]. Interestingly, the interaction of PAT-SM6 with csGRP78 was shown to induce the accumulation of intracellular lipids, which, in turn, induced apoptosis in 23132/87 gastric carcinoma cell lines [80]. PAT-SM6 has previously been evaluated in the treatment of multiple myeloma [81]. It was shown that PAT-SM6 alone induced cytotoxicity in primary and cultured myeloma cells [81]. Moreover, it was demonstrated that PAT-SM6 also contributes to the complement-dependent cytotoxicity of primary and cultured multiple myeloma cell lines [81]. Promising phase I clinical trial results have indicated that PAT-SM6 is safe and well-tolerated in patients with relapsed or refractory multiple myeloma, with satisfactory pharmacokinetic parameters [82]. Furthermore, PAT-SM6 was recently shown to have synergistic effects with existing anti-multiple myeloma combination therapies such as bortezomib and lenalidomide.

Unlike MAb, scFvs are engineered antibodies that contain a single variable light and heavy chain connected by a flexible peptide linker [83]. Furthermore, an advantage of scFv over MAb is that scFvs can be generated in recombinant protein bacterial expression systems, allowing for the rapid production of scFvs without the need for expensive hybridomas [83]. Anti-GRP78 scFvs conjugated to quantum dot (Qdot) nanobeads have effectively been used to fluorescently label MDA-MB-231 breast cancer and LNCaP prostate cancer cells, which express csGRP78. Intriguingly, incubation of the anti-GRP78 scFv/Qdot conjugates induced apoptosis in MDA-MB-231 cells. In addition, anti-GRP78 scFv/Qdot conjugates attenuated MDA-MB-231 tumor growth, compared with unlabelled nanobeads [84]. Although these findings indicate that scFvs which target GRP78 are a promising diagnostic and therapeutic tool, further investigation of the mechanisms by which the anti-GRP78 scFv/Qdot conjugate induces apoptosis and inhibits tumor growth is required. GSF3, an scFv that specifically targets the C-terminal domain of GRP78, was recently identified using a ribosome display panning method [85]. Given its specificity for the C-terminus of GRP78, GSF3 may exhibit similar anti-tumorigenic effects to the MAb that target C-terminal GRP78.

### 4.4. Clinical Trials, Limitations and Challenges

PAT-SM6, an anti-GRP78 monoclonal antibody used in a Phase I trial, was well-tolerated with modest clinical benefit in multiple myeloma (NCT04421820). Furthermore, IT-139 (also known as NKP1339, BOLD-100), a ruthenium-based anti-cancer compound that targets GRP78, was evaluated in a Phase I trial (NCT01415297) in forty-six patients with solid tumors. Burris et al. reported that overall IT-139 monotherapy was well tolerated, with modest anti-tumor effects [86]. The results from these initial clinical studies suggest that therapies targeting GRP78 have clinical value and require continued investigation. Currently, a Phase I trial (NCT04421820) is recruiting participants to evaluate IT-139 in combination with chemotherapy. Although csGRP78 is a promising therapeutic target in cancer treatment, there are several limitations, such as potential off-target effects. Several studies have reported the expression and function of csGRP78 in non-cancer pathologies, including atherosclerosis, thrombotic disease, rheumatoid arthritis, and diabetic nephropathy, thereby potentially complicating the utility of csGRP78-based therapies in cancer patients with co-morbidities [87,88,89].

## 5. Targeting Cell Surface GRP78/Anti-GRP78 Autoantibody Complex

As previously discussed, the binding of anti-GRP78 autoantibodies to the N-terminal region of csGRP78 promotes malignant tumor growth and metastasis. Hence, disrupting the csGRP78/anti-GRP78 autoantibody complex may lead to novel therapeutics [44,67]. Our previous studies have demonstrated that the low-molecular-weight heparin, enoxaparin, blocked the binding of the anti-GRP78 autoantibody to csGRP78, resulting in the attenuation of DU145 xenograft growth in mice [44]. Mechanistically, because a heparin-binding domain has been identified near the anti-GRP78 autoantibody epitope on csGRP78 [43], heparin-like molecules may function as competitive antagonists that disrupt the binding of autoantibodies to csGRP78 [43]. An alternative strategy to disrupt the anti-GRP78 autoantibody complex is to identify small molecules that bind to GRP78 and hinder its interaction with the autoantibody. Furthermore, MAb that bind to alternative epitopes on csGRP78 may interfere with pro-tumorigenic GRP78 N-terminal antibodies. Interestingly, antibodies targeting the C-terminal region of GRP78 have been shown to inhibit the interaction of the N-terminal-specific anti-GRP78 antibody in mice [67]. Further investigations should be conducted to examine whether a similar effect occurs with cancer-patient-derived anti-GRP78 autoantibodies. 

## 6. Summary

Since its initial discovery, GRP78 has been well-established as the master regulator of ER-resident polypeptide folding by activating the UPR and downstream pathways that promote cellular apoptosis and autophagy in eukaryotes. Our understanding of GRP78 has evolved in recent years, recognizing its multifaceted role beyond its mere regulation of protein folding in the ER. GRP78 has been shown to localize to the cell surface and its influence on tumor growth is evident. Hence, it is of utmost importance for investigations to continue to expand our understanding of the role of GRP78 on the cell surface in order to develop novel therapeutic modalities to combat cancer cell progression.

## Data Availability

Not applicable.

## Abbreviations

α_2_M, α_2_-macroglobulin; AML, acute myeloid leukemia; ATF6, activating transcription factor 6; ATP, adenosine triphosphate; BiP, binding immunoglobulin protein; BMTP-78, bone metastasis targeting peptidomimetic-78; CD44v, an isoform of CD44; csGRP78, cell surface GRP78; DCD, Dermcidin; DNAJC3, DnaJ homolog subfamily C member 3; ER, endoplasmic reticulum; FADD, Fas-associated death domain; GIV, G_α_-interacting vesicle-associated protein; GPI, glycosylphosphatidylinositol; GRP78, 78 kDa glucose-regulated protein; HNSCC, head and neck squamous cell carcinoma; IP_3_, inositol triphosphate; IRE1α, inositol-requiring protein 1α; KDELR, KDEL receptor; MAb, monoclonal antibodies; MTJ-1, murine Dna-J-like transmembrane protein; Par-4, prostate apoptosis response 4; PERK, protein kinase RNA-like ER kinase; rGRP78, recombinant GRP78; SPECT, single-photon emission computerized tomography; SRC, proto-oncogene tyrosine–protein kinase; TF, tissue factor; TGFβ, tumor growth factor β; TRAIL, TNF-related apoptosis-inducing ligand; UPR, unfolded protein response.
